# Academic training selectively shapes the neural pathways mediating sex/gender differences in cognition

**DOI:** 10.1186/s13293-026-00963-0

**Published:** 2026-08-06

**Authors:** Sandrine Cremona, Laure Zago, Christophe Tzourio, Marc Joliot

**Affiliations:** 1 Univ. Bordeaux, CNRS, IMN UMR 5293, F-33000 Bordeaux, France; 2 Univ. Bordeaux, INSERM, BPH U1219, F-33000 Bordeaux, France; 3 Univ. Bordeaux, CEA, CNRS, IMN UMR 5293, F-33000 Bordeaux, France

**Keywords:** Sex, Gender, Brain functional connectivity, Cognition, Higher education

## Abstract

**Background:**

Sex and gender (S/G) differences in cognitive performance are well documented, yet how they relate to brain organization and interact with educational experience during early adulthood, a period of ongoing cognitive maturation, remains unclear. University training exposes young adults to distinct cognitive and social demands, providing a natural context to test whether brain functional connectivity (FC) mediates S/G cognitive differences in an education-dependent manner.

**Methods:**

The cohort included students enrolled in declarative fields (formalized, context-independent knowledge, e.g., STEM, law, business administration, philosophy) or situated fields (knowledge embedded in social practice, e.g., psychology, education, medicine). Individual FC networks were derived using the GINNA atlas; edges significantly differing between S/G groups were identified and aggregated into sex-differential nodal FC indices (sdNFIs). Moderated mediation models tested whether academic training fields shape the contribution of these sdNFIs to S/G differences in cognitive performance. Additional exploratory analyses tested whether effects varied across academic stages by including study level as a second moderator.

**Results:**

Baseline S/G differences in cognitive performance and sdNFIs were preserved across academic training. Yet sdNFI-mediated pathways linking S/G to cognitive performance varied strongly by knowledge domain. In declarative fields, sdNFIs spanning parietal and medial temporal networks supported male advantages in visuospatial and arithmetic tasks. In situated fields, sdNFIs spanning medial prefrontal and temporo-parietal networks supported female advantages in socio-emotional and verbal tasks. Study-level analyses revealed distinct profiles of sdNFI mediation across tasks. sdNFI-mediated effects were observed primarily at later academic stages (Y2 +) for arithmetic fact retrieval, mental rotation, and emotion recognition, whereas they were present at Y1 but absent at later stages for verbal list learning.

**Conclusions:**

These findings demonstrate that academic training selectively shapes FC-cognition relationships in a S/G-specific manner, with declarative and situated knowledge domains recruiting distinct, context-sensitive networks to support performance. By guiding the recruitment of functional networks to meet specific cognitive demands, higher education may constitute a formative period during which the brain architecture supporting S/G cognitive differences is dynamically reorganized.

**Plain English summary**

Men and women often differ in certain cognitive skills: women tend to perform better in tasks involving language and recognizing emotions, while men tend to show advantages in spatial reasoning and arithmetic. These differences are well established, but their origins, biological, environmental, or both, remain debated.

In this study, we asked whether the type of university education a person follows influences how the brain supports these cognitive differences. We compared students enrolled in fields centered on formal knowledge, such as STEM, law, or philosophy, with students in fields where learning is deeply embedded in human interaction and social practice, such as psychology, education, or medicine.

We found that men and women showed similar cognitive differences regardless of their field of study. However, the brain pathways supporting these differences varied depending on the type of training. In formal knowledge fields, brain connections in spatial and memory-related regions supported male advantages in arithmetic and visuospatial tasks. In socially oriented fields, brain connections in regions involved in language and social processing supported female advantages in verbal memory and emotion recognition. Examining these relationships across academic stages revealed different patterns: some brain-performance associations emerged with university training, whereas others faded after the first year, highlighting how flexibly the brain adapts to educational experience.

These findings suggest that higher education does not eliminate cognitive differences between men and women, but shapes how these differences are reflected in the brain. Understanding how education interacts with brain development may help design learning environments that better support all students.

**Supplementary Information:**

The online version contains supplementary material available at https://doi.org/10.1186/s13293-026-00963-0.

## Highlights

- Sex and gender (S/G) differences in cognitive performance and functional connectivity (FC) are preserved across declarative and situated academic training fields, indicating that the education domain does not alter baseline S/G cognitive profiles.
- The FC pathways mediating S/G cognitive differences vary strongly as a function of knowledge domain, revealing experience-dependent network recruitment rather than fixed brain-behavior relationships.
- In declarative fields, parietal and medial temporal networks support male advantages in visuospatial and arithmetic tasks; in situated fields, medial prefrontal and temporo-parietal networks support female advantages in socio-emotional and verbal tasks.
- Study-level analyses revealed heterogeneous profiles of FC mediation, with effects emerging at later academic stages for some cognitive tasks and attenuating for others, consistent with experience-dependent reorganization of network-cognition relationships.

**Supplementary Information:**

The online version contains supplementary material available at https://doi.org/10.1186/s13293-026-00963-0.

## Background

Sex and gender (S/G) differences in human cognition represent one of the most replicated yet contested findings in cognitive neuroscience. At the population level, women consistently outperform men in verbal and socio-emotional domains, while men show advantages in visuospatial and numerical tasks [1, 2]. Despite the robustness of these patterns, their interpretation remains unresolved: whether they primarily reflect biological differentiation, sociocultural experience, or their interaction is still debated [3]. This debate has placed S/G at the forefront of contemporary research priorities, as underscored by guidelines such as NIH Sex as a Biological Variable (SABV) and SAGER, which mandate the systematic integration of S/G into study design, analysis, and reporting [4, 5].

Addressing these questions requires tools capable of capturing individual variation in brain organization at scale. The rapid expansion of large-scale neuroimaging cohorts has brought unprecedented statistical power to the study of brain-behavior relationships, enabling robust, population-level investigations of individual differences in cognition [6, 7]. Among available neurobiological measures, functional connectivity (FC) offers a particularly informative window into the distributed network organization supporting cognitive performance. Intrinsic FC reliably captures coordinated activity across distant brain regions [8], demonstrates strong test–retest reliability and generalizability across independent cohorts [9], making it especially well-suited for characterizing individual differences in cognition.

Emerging evidence links S/G cognitive differences to large-scale functional network organization: FC of the left posterior cingulate cortex has been shown to mediate S/G differences in general cognitive performance [10], visual and salience network connectivity has been implicated in S/G differences in visuospatial abilities in young adults [11], and deep learning approaches have identified replicable, sex-specific FC patterns predictive of cognitive performance across independent cohorts [12]. However, such findings are typically derived from designs in which participants are classified into binary male/female categories. Although these categories are often interpreted as reflecting biological sex, several authors have argued that the resulting group differences may also capture sociocultural influences associated with gendered experiences, making it difficult to disentangle biological and social contributions to observed effects [13, 14, 15, 16]. Critically, this operationalization forecloses the possibility of examining whether S/G-related brain-behavior relationships are themselves shaped by environmental context, a question that remains largely unaddressed [16].

Among the structured environmental contexts available to test this hypothesis, higher educational experience represents a sustained source of environmental variation that shapes cognitive development not only through the accumulation of domain-specific knowledge, but through the particular blend of cognitive demands, learning strategies, and social engagement that characterizes different fields of study. This period coincides with continued maturation of large-scale association networks supporting higher-order cognition [17, 18], and learning and expertise are known to reshape FC within and between these distributed networks, reflecting experience-driven tuning of task-relevant circuits [19, 20]. Higher education therefore offers a privileged naturalistic context to examine whether the brain-behavior pathways underlying S/G cognitive differences are stable across educational environments or selectively modulated by the nature of academic training, and, crucially, to ask what dimension of that training matters.

Here, we leverage this context to test whether educational field shapes FC-mediated S/G cognitive differences in young adults. Rather than following conventional thematic taxonomies, we organized fields of study according to the epistemic nature of the knowledge they mobilize, i.e., the degree to which knowledge is formalized and context-independent versus embedded in action and social practice. We distinguished two broad categories of university training: *declarative fields* (e.g., STEM, law, business administration, philosophy), in which learning is primarily organized around the acquisition of decontextualized knowledge, and *situated fields* (e.g., psychology, education, medicine), in which knowledge is systematically integrated with social interaction and real-world practice as core vectors of learning and competence [21, 22]. Critically, this distinction does not imply an opposition between two mutually exclusive knowledge types, declarative knowledge is present in both, but reflects a gradient in the degree to which training foregrounds socially embedded, practice-oriented cognition as a target of competence in its own right [23]. We derived individual FC networks and computed sex-differential connectivity indices to test whether the functional networks mediating task performance vary with this educational gradient, and whether such modulation differs between male and female students. This approach allows us to reframe the question of S/G cognitive differences within a dynamic system framework [24]: rather than asking whether they are biologically stable or environmentally shaped, we ask through which experience-dependent network pathways they are expressed, and how these pathways are selectively organized by the nature of academic training.

## Methods

### Participants

Data were drawn from the Internet-based Students Health Research Enterprise (i-Share), a large prospective cohort of French university students aged 18–35 years designed to investigate student health (https://research.i-share.fr/). Participants enrolled voluntarily and completed a mandatory baseline questionnaire, along with an optional module assessing cognitive functioning using a computerized cognitive battery. The i-Share study was approved by the French National Commission on Informatics and Liberties and conducted in accordance with the Declaration of Helsinki. The neuroimaging protocol was approved by the Comité de Protection des Personnes Sud-Ouest et Outre-Mer (local ethics committee CPP SOOMIII; agreement 2015-A00850-49). All participants provided written informed consent.

A subset of participants also took part in the neuroimaging arm of the project, aimed at characterizing neuroanatomical maturation in healthy young adults (MRi-Share [25]). MRi-Share participants underwent a 15-min eyes-closed resting-state fMRI scan, followed by completion of the Resting-State Questionnaire (ReSQ 2.0; 24-item self-report) assessing resting-state thought profiles (RSTPs [26]). Native French speakers with usable MRI data were included in the FC analyses (mean age = 22.0 ± 2.25 years; 72% women; n = 1,771). Samples used for cognitive performance analyses were defined as the intersection between the FC sample and each cognitive task, resulting in sample sizes ranging from 1,158 to 1,261 participants, depending on the test. Sex was self-reported as male or female. Because differences between these categories may reflect both biological and sociocultural influences that cannot be distinguished with the available data, we refer to this variable throughout the manuscript as sex and gender (S/G).

### Academic training

Knowledge domain was used as a proxy for academic training, defined by the primary epistemic orientation of each field, i.e., the type of knowledge emphasized and the processes by which it is acquired and applied. Fields were classified according to the degree to which knowledge is formalized and context-independent versus embedded in action and social practice. We distinguished *declarative fields* (e.g., STEM, law, business administration, philosophy), which focus on content knowledge, from *situated fields* (e.g., psychology, education, medicine), where content knowledge is integrated with engagement in human behavior and socially situated cognition [21, 23]. Importantly, the proportion of male and female participants did not differ between the two academic domains in the present sample. The distribution of S/G across academic domains and cognitive task subsamples is reported in Additional file 1, with men representing 25–28% of participants across subsamples.

### Cognitive assessment

Cognitive tasks were selected in domains that have repeatedly demonstrated S/G differences in prior literature: emotion recognition and verbal list learning in women, mental rotation, visuospatial reasoning, and arithmetic performance in men [1, 27]. Two arithmetic tasks were included to contrast automatic fact retrieval with multi-step calculation, which engages a broader range of solution strategies [28]. Four tests were collected via the voluntary online survey of the i-Share study, titled “Comment ça va, les neurones” ? (hereafter referred to as online), while two tests were completed on a laboratory computer on-site after the resting-state fMRI session (hereafter referred to as on-site).

Emotion recognition (N = 1261) was assessed on-site using the short version of the Geneva Emotion Recognition Test (GERT-S) [29], which evaluates the ability to identify others’ emotions from prosody and facial expressions. Participants viewed short videos of male and female actors expressing one of 14 emotions (Amusement, Joy, Surprise, Disgust, Sadness, Despair, Relief, Anxiety, Anger, Pleasure, Fear, Irritation, Interest, Pride), with each emotion presented three times. Performance corresponded to the total number of correct responses across the 42 presentations.

Verbal list learning (N = 1200) was measured on-site using a short version of the Rey Auditory Verbal Learning Test (RAVLT) [30]. Participants listened to a list of 15 concrete nouns and recalled as many words as possible immediately (immediate recall, trial 1) by typing them on the keyboard. After a 20-min delay, corresponding to the completion of the GERT task, they recalled the words again (delayed recall, trial 2). Because the verbal learning task included only one encoding trial and one delayed recall trial, immediate and delayed recall scores were combined into a composite verbal memory score. This approach was adopted to provide a more robust estimate of overall verbal memory performance and is consistent with previous work using the same dataset [31].

Arithmetic fact retrieval (N = 1245) was assessed online using a multiplication verification task comprising 36 single-digit multiplication problems (e.g., 4 × 3 = 16), presented sequentially for 2.5 s each, with a 0.5 s inter-stimulus interval. Half of the problems were incorrect. Participants indicated whether the result was correct or incorrect using the left/right arrow keys (left = correct, right = incorrect).

Mental rotation (N = 1244) was assessed online using a shortened version of the Vandenberg and Kuse test [32]. Participants identified the two 3D figures among four options that matched a target figure rotated in space. Performance was scored as correct only when both matching figures were selected.

Visuospatial reasoning (N = 1158) was evaluated online using a shortened version of the Raven’s Progressive Matrices [33]. Each item consisted of a matrix of nine visual patterns with one missing element, which participants completed by selecting the correct option among eight alternatives.

Calculation (N = 1193) was measured online with six complex arithmetic problems involving multiplications, additions, and subtractions (e.g., 581 – 126 = ?). Problems were presented sequentially for up to 30 s each, with a 0.5 s inter-stimulus interval. Participants mentally computed each result and entered it using the keyboard.

### Test of S/G differences in cognitive performance

S/G cognitive differences were evaluated using linear models for each task, adjusting for age, intracranial volume, and RSTPs. Interactions between S/G and knowledge domain were tested using Type III sums of squares and were not significant; therefore, adjusted mean differences were estimated from models including S/G as a main effect only. Ninety-five percent confidence intervals (CIs) were obtained by non-parametric bootstrap, and statistical significance was assessed using false discovery rate (FDR)-corrected *P* values. In addition, standardized effect sizes (Cohen’s *d*) for S/G differences were computed on scaled raw scores, without covariate adjustment, to allow direct comparison with prior literature.

### Resting-state fMRI acquisition and preprocessing

Acquisition and preprocessing are described in detail in [25]. All neuroimaging data were acquired between November 2015 and November 2017 on a Siemens 3 T Prisma scanner equipped with a 64-channel head coil. Resting-state fMRI data were collected during a single 15-min run using a multiband EPI sequence (2.4 mm isotropic voxels; TR = 850 ms; TE = 35 ms; flip angle = 56°; multiband factor = 6), yielding 1,054 volumes per participant. Participants were instructed to keep their eyes closed, remain awake, relax, and minimize movement.

Preprocessing consisted of distortion correction, registration of resting-state fMRI data to anatomical space, temporal band-pass filtering (0.01–0.1 Hz), and normalization to MNI space (2 mm isotropic). Motion-related variance was removed by regression of movement parameters. Preprocessing was performed using FSL v5.0.1059 and AFNI v10.0.0560 within a Singularity container.

### Estimation of sex-differential nodal FC indices (sdNFIs)

Individual functional connectomes were derived using the Groupe d’Imagerie Neurofonctionnelle Network Atlas (GINNA). The three steps underlying the GINNA atlas, (i) individual independent component analysis (ICA) for each participant, (ii) classification of ICs into classes, and (iii) atlas construction, are described in detail in [34]. The non-overlapping version of the GINNA atlas includes 439 brain regions organized into 32 cortical functional networks, yielding 496 Fisher-z-transformed edges per participant (32 × 31 / 2). These networks can be classified into seven modules: social and communication, self-awareness, visual, somatomotor, cognitive flexibility, performance monitoring, and executive planning [35]. Edge-wise FC values were normalized by the mean within-group correlation to control for baseline S/G differences in resting-state FC, thereby isolating FC variance associated with cognitive performance rather than resting-state sexual dimorphism.

Edge pre-selection identified FC features potentially associated with S/G. FC values across all edges were estimated simultaneously using a linear mixed model including S/G, covariates (age, intracranial volume, RSTPs), a random intercept per participant, and random slopes of S/G for each edge. Nested cross-validation (10 repetitions × 5 outer folds) ranked edges by absolute slope, and stability selection (frequency ≥ 0.8) with Bonferroni-corrected  *P *< 0.05, yielding 72 edges. These were entered into a LASSO logistic regression (λ = 1, selected to maximize sparsity) predicting S/G, adjusting for covariates; 19 edges with non-zero coefficients were retained. From the LASSO subset, we computed sex-differential nodal FC indices (sdNFIs), calculated for each node as the aggregate of FC connections showing significant S/G differences. This procedure yielded two sdNFIs in women (sdNFIs 3, 28) and seven in men (sdNFIs 1, 3, 5, 7, 15, 22, 28).

### Statistical analyses

**Fig. 1:**
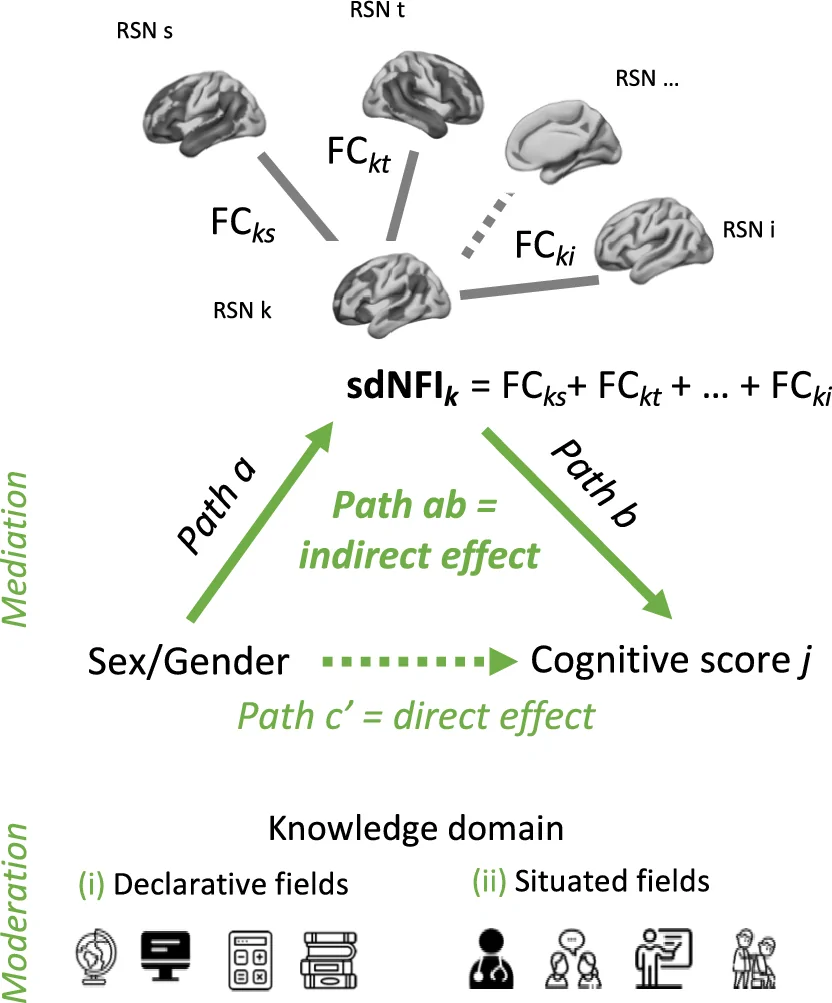
Moderated mediation framework (Hayes’ Model 59). The indirect effect of S/G on cognitive performance via sdNFI (sex-differential nodal FC index) was estimated separately for each level of the knowledge domain moderator: declarative fields (formalized, context-independent knowledge) and situated fields (knowledge embedded in social practice)

Moderated mediation analyses were conducted to test whether S/G-specific sdNFI mediates the relationship between S/G and cognitive performance, and whether this mediation depends on academic training fields (Fig. 1).

For each S/G-advantaged cognitive task, we paired it with all relevant S/G-specific sdNFIs (e.g., two female sdNFIs for two female-advantaged tasks = four models; seven male sdNFIs for four male-advantaged tasks = 28 models). Each model estimates: (i) the direct effect of S/G on cognition (c’ path), (ii) the indirect effect of S/G on cognition via sdNFI (ab path), and (iii) whether the strength of this indirect effect varies depending on the moderator, i.e., knowledge domain (declarative vs. situated fields). All models included age, intracranial volume, and RSTPs as covariates. Indirect effects were estimated via a bootstrap approach using PROCESS Model 59, a moderated mediation model with one mediator and one categorical moderator [36], with 5,000 bootstrap samples. Significance of indirect effects was determined using bias-corrected bootstrap CIs, with mediation considered significant when the CI excluded zero. FDR correction was applied to the corresponding direct-effect tests to account for multiple comparisons. Corrections were performed separately for male- and female-advantaged models (28 and 4 models, respectively), given the a priori sex-specificity of each sdNFI. To facilitate interpretation of the mediation models, we additionally report the total effect (c path) of S/G on each cognitive outcome. This total effect was estimated using separate linear regression models specified with the same covariate structure as the mediation models. It is important to note that, in the context of multiple mediation models estimated using PROCESS Model 59, the total, direct, and indirect effects are not expected to be algebraically additive. This is due to differences in estimation procedures across equations and the partitioning of variance in multi-mediator frameworks. Consequently, the total effect is reported solely as a descriptive reference for the overall association between S/G and cognitive performance, and should not be interpreted as a quantity decomposable into direct and indirect components.

To examine the directional specificity of the observed FC-cognition relationships, complementary models were estimated reversing the mediator and outcome: cognitive performance as mediator and sdNFI as outcome, while retaining knowledge domain as moderator. This allows testing whether the observed associations are specific to the direction from FC to cognition, or whether reciprocal influences exist. All models included age, intracranial volume, and RSTPs as covariates, and indirect effects were estimated using the same bootstrap procedure as in the primary analyses (5,000 resamples).

To investigate whether the moderated mediation pathway was already present at entry into higher education or emerged with subsequent academic exposure, complementary exploratory analyses incorporated study level as an additional moderator. Specifically, conditional indirect effects were estimated separately for first-year students (representing approximately 29–31% of participants in each subsample) and students at more advanced stages of university training (Year 2 and above) using structural equation modeling implemented in the lavaan package for R. Moderation effects were specified on both the a path (S/G → sdNFI) and b path (sdNFI → cognition), including interaction terms between S/G, sdNFI, knowledge domain, and study level. Conditional indirect effects (ab path) were computed manually from model-estimated coefficients within each study-level group and knowledge domain. Statistical inference relied on a nonparametric bootstrap procedure with 5,000 resamples, generating empirical sampling distributions for each conditional indirect effect. Point estimates correspond to the standardized indirect effect (Z-Score), and 95% CIs were derived from the percentile bootstrap distribution. Effects whose CIs did not include zero were considered statistically significant. Bootstrap-derived two-sided *P *values (*P* BOOT) were computed from the empirical bootstrap distributions and are reported for descriptive purposes. These analyses were conducted only for sdNFI-cognition pairs that showed significant moderated mediation effects in the primary analyses.

Statistical analyses were performed using R (version 4.0.0; R Core Team, Vienna, Austria) within the RStudio integrated development environment (version 2025.9.2.418; RStudio Team).

## Results

### S/G differences in cognitive performance are preserved across knowledge domains

**Fig. 2:**
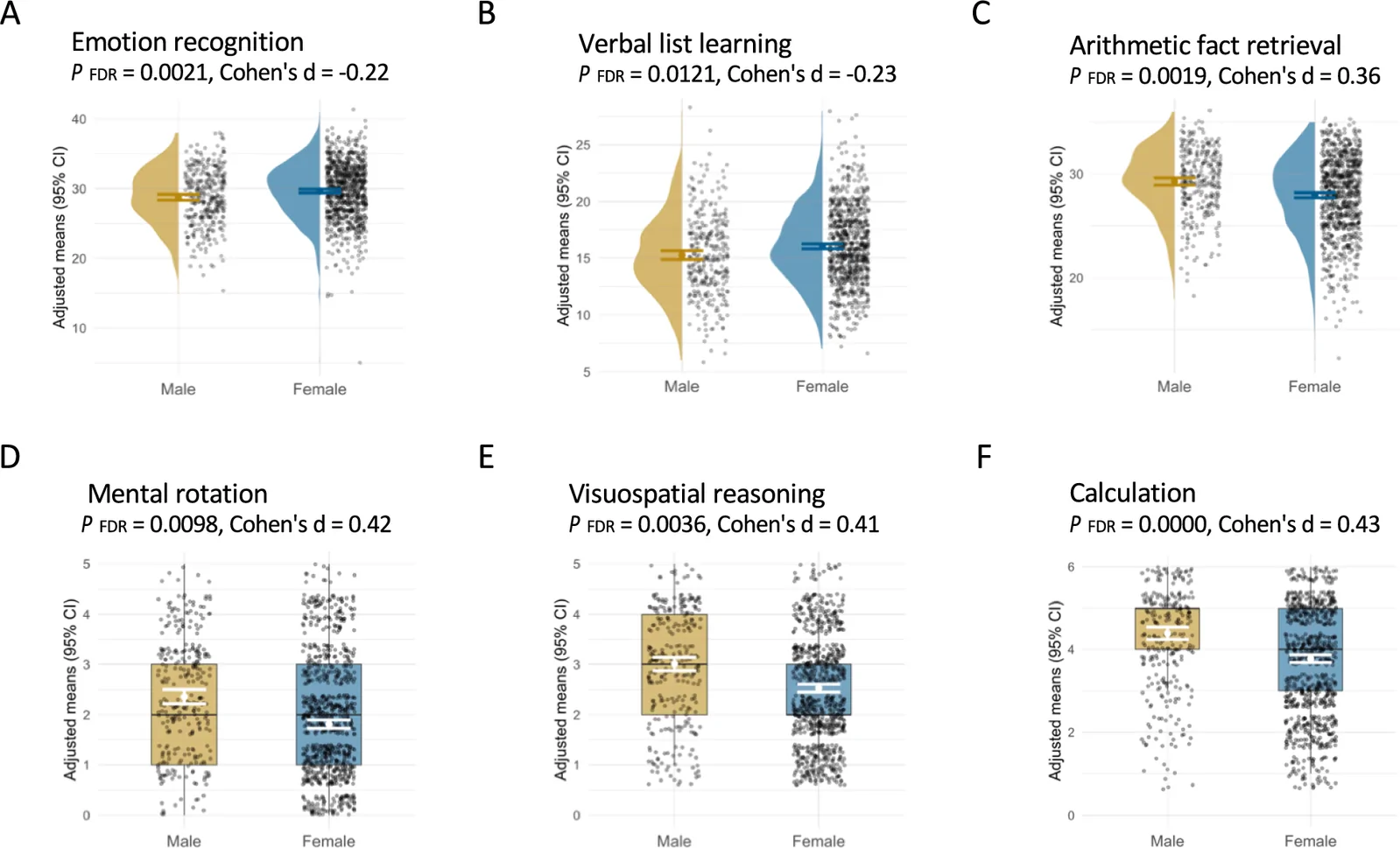
S/G differences in cognitive performance. Panels **A**-**F** show the distribution of cognitive performance for women and men in tasks exhibiting significant S/G differences. Points and error bars indicate adjusted means with 95% CIs; exact mean values and CIs are reported in Table 1. FDR *P* values and Cohen’s *d* effect sizes are indicated for each task

Consistent with prior literature, women showed higher mean performance in emotion recognition and verbal list learning, whereas men showed higher mean accuracy in arithmetic fact retrieval, mental rotation, visuospatial reasoning, and calculation (Table 1; Fig. 2).

**Table 1:** S/G differences in cognitive performance

	**Mean M**	**Mean F**	**M - F**	**CI95% low**	**CI95% high**	***P* FDR**	**Cohen’s d**	**S/G × Knowledge domain**	
								**F**_**(1)**_	***P***
**Emotion recognition**									
*N* = 1261	28.56	29.77	-1.22	-1.8600	-0.5798	0.0004	-0.22	0.01	0.9149
**Verbal list learning**									
*N* = 1200	15.30	16.00	-0.70	-1.2585	-0.1363	0.0121	-0.23	2.50	0.1138
**Arithmetic fact retrieval**									
*N* = 1245	29.02	28.06	0.97	0.4171	1.4950	0.0019	0.36	0.90	0.3419
**Mental rotation test**									
*N* = 1244	2.17	1.88	0.28	0.0628	0.4978	0.0098	0.42	0.87	0.3509
**Visuospatial reasoning**									
*N* = 1158	2.87	2.58	0.29	0.0942	0.4853	0.0036	0.41	0.52	0.4691
**Calculation**									
*N* = 1193	4.34	3.79	0.55	0.3286	0.7805	0.0000	0.43	0.68	0.4079

The observed Cohen’s *d* effect sizes (Table 1) fell within previously reported ranges: meta-analyses consistently report small female advantages in emotion recognition (Cohen’s ∣*d*∣ ≈ 0.24) [27] and verbal memory (∣*d*∣ ≈ 0.28) [37], whereas small to moderate male advantages have been reported for spatial abilities (∣*d*∣ ≈ 0.37) [38] and mental arithmetic performance (∣*d*∣ ≈ 0.47) [39]. Together, the magnitude and direction of the observed S/G differences closely match large-scale meta-analytic findings, supporting both the representativeness of the cohort and the robustness of the behavioral measures. S/G differences in cognitive performance were preserved across academic training, with no significant S/G × knowledge domain interaction (Table 1). Baseline S/G differences in cognitive performance were thus independent of knowledge domain.

### S/G differences in FC are distributed across multiple functional networks

Individual functional connectomes were constructed using the GINNA atlas [34], comprising 32 functional networks, resulting in 496 Fisher-z-transformed edges per participant (32 × 31 / 2). Edge-wise linear mixed-effects models were then used to identify edges potentially associated with S/G. Stability selection (frequency ≥ 0.8, Bonferroni-corrected *P* < 0.05) retained 72 edges, which were entered into a LASSO regression. Nineteen edges with non-zero LASSO coefficients were used to compute sdNFIs. An additional figure file shows this in more detail [see Additional file 2].

**Fig. 3:**
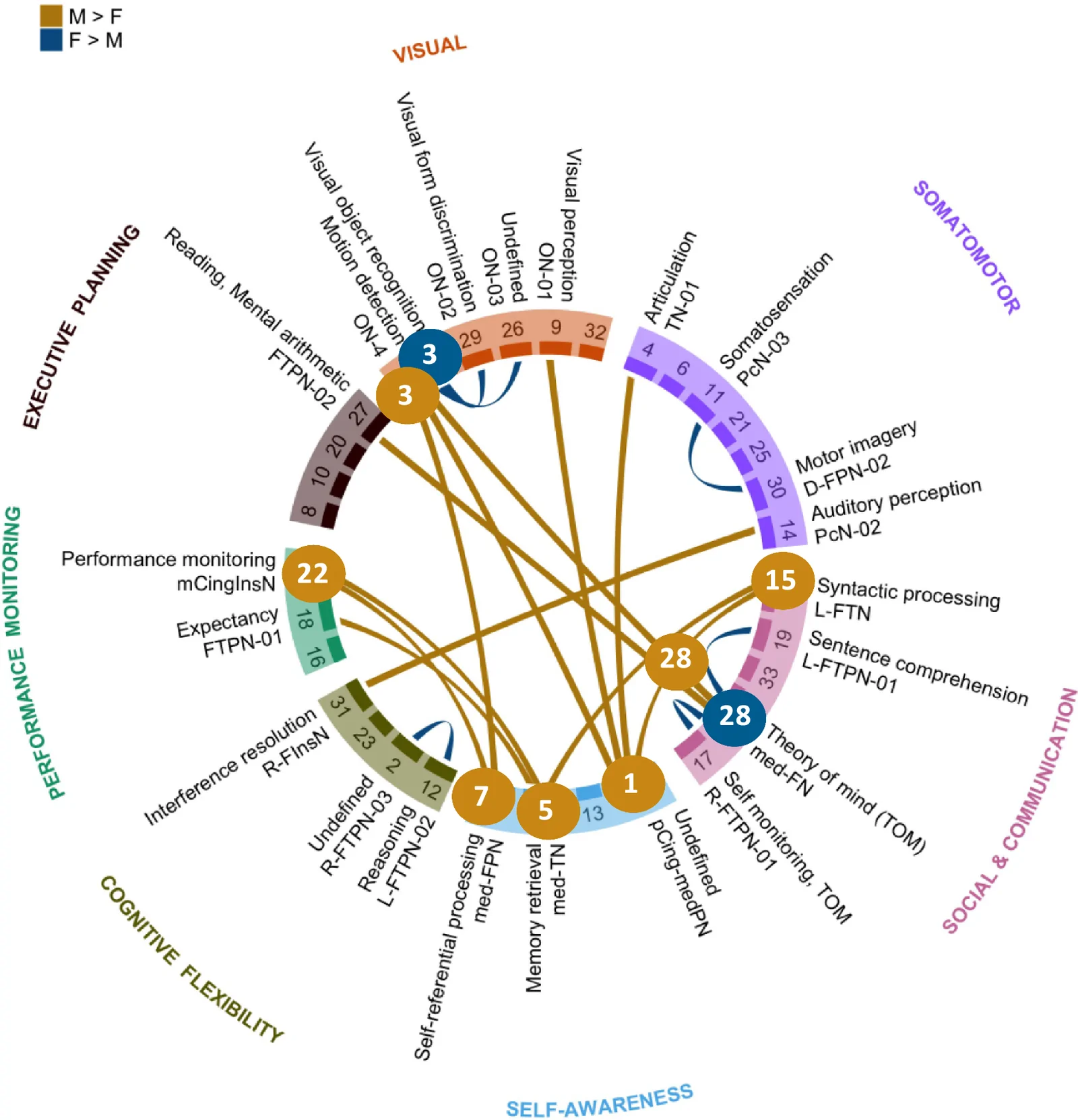
The 19 FC edges significantly differing between men and women. Edges were retained after edge-level mixed modeling with stability selection (frequency > 0.8; *P* BON < 0.05) followed by LASSO. Numbers denote GINNA networks (1-32) and their cognitive and anatomical characterization. Track colors indicate modules. Link colors indicate M > F (beige) and F > M (blue). sdNFIs (sum of edge weights sharing the same seed) are highlighted with circles. Abbreviations: a/m/pCing = anterior/middle/posterior cingulate; F = frontal; Ins = insular; L = left; med = medial; N = network; O = occipital; P = parietal; R = right; T = temporal

Two sdNFIs were identified in women (sdNFIs 3 and 28) and seven in men (sdNFIs 1, 3, 5, 7, 15, 22, and 28) (Fig. 3; an additional table file shows this in more detail [see Additional file 3]). In women, sdNFI-3 was primarily intra-visual, whereas sdNFI-28 spanned into the social and communication module. In men, sdNFIs were distributed across five modules: performance monitoring, self-awareness, social and communication, cognitive flexibility, and visual. This asymmetry is consistent with prior evidence of greater inter-module FC integration in men and stronger intra-module cohesion in women [40, 41,42].

### Academic training shapes sdNFI mediation of S/G cognitive differences

To test whether FC mediates S/G cognitive differences as a function of academic training, we estimated separate moderated mediation models [36]. For each S/G-advantaged cognitive task, one model was run per S/G-specific sdNFI (e.g., two female sdNFIs for two female-advantaged tasks = four models; seven male sdNFIs for four male-advantaged tasks = 28 models). Indirect effects of S/G on cognition via sdNFIs were assessed in declarative vs. situated fields within the same model. Significant indirect effects emerged selectively as a function of knowledge domain, task, and sdNFI (Table 2 and Additional file 4). Knowledge domains (i.e., declarative *vs* situated) consistently modulated the coupling between sdNFI and cognitive performance, rather than the association between S/G and sdNFI, indicating that academic training fields selectively shapes how networks support task-specific cognition without significantly altering baseline S/G differences in FC.

**Table 2:** Significant sdNFI-mediated S/G differences in cognition across academic training*

		**Knowledge domain (Moderator)**	
**Outcome**	**Mediator**	**Declarative fields**	**Situated fields**
***Female advantaged-performance***			
Emotion recognition		c**: -1.211 [-2.035, -0.377]	c**: -1.183 [-2.148, -0.243]
	sdNFI-3	**a: -0.362 [-0.679, -0.045]** **b: 0.189 [0.012, 0.367]** **ab: -0.069 [-0. 180, -0.000]** **c’: -1.136 [-1.901, -0.372]**	**a: -0.533 [-0.883, -0.182]** **b: 0.293 [0.092, 0.495]** **ab: -0.156 [-0.320, -0.030]** **c’: -0.984 [-1.834, -0.135]**
	sdNFI-28	**a: -0.420 [-0.655, -0.185]** b: 0.008 [-0.233, 0.249] ab: -0.004 [-0.122, 0.113] **c’: -1.230 [-2.001, -0.459]**	**a: -0.640 [-0.900, -0.380]** **b: 0.447 [0.177, 0.717]** **ab: -0.286 [-0.519, -0.105]** c’: -0.858 [-1.718, 0.001]
Verbal list learning		c**: -0.790 [-1.502, -0.063]	c**: -0.562 [-1.403, 0.266]
	sdNFI-28	**a: -0.485 [-0.728, -0.242]** b: 0.106 [-0.316, 0.103] ab: -0.051 [-0.171, 0.052] **c’: -0.926 [-1.599, -0.253]**	**a: -0.643 [-0.910, -0.377]** **b: 0.263 [0.032, 0.494]** **ab: -0.169 [-0.358, -0.021]** c’: -0.100 [-0.841, 0.641]
***Male advantaged-performance***			
Mental rotation		c**: 0.377 [0.076, 0.685]	c**: 0.190 [-0.101, 0.480]
	sdNFI-5	**a: 0.862 [0.271, 1.453]** **b: 0.038 [0.003, 0.072]** **ab: 0.033 [0.003, 0.079]** **c’: 0.337 [0.071, 0.602]**	**a: 0.852 [0.251, -1.453]** b: 0.014 [-0.022, 0.050] ab: 0.012 [-0.019, 0.048] c’: 0.194, [-0.077, 0.464]
	sdNFI-15	**a: 0.478 [0.117, 0.840]** **b: 0.062 [0.004, 0.119]** **ab: 0.029 [0.001, 0.076]** **c’: 0.339 [0.074, 0.605]**	**a: 0.565 [0.198, 0.933]** b: 0.030 [-0.028, 0.088] ab: 0.017 [-0.016, 0.056] c’: 0.188 [-0.083, 0.458]
Visuospatial reasoning		c**: 0.435 [0.148, 0.703]	c**: 0.150 [-0.125, 0.425]
	sdNFI-5	**a: 0.900 [0.283, 1.516]** **b: 0.035 [0.004, 0.067]** **ab: 0.032 [0.003, 0.074]** **c’: 0.331 [0.087, 0.574]**	**a: 0.835 [0.222, 1.448]** b: 0.013 [-0.019, 0.045] ab: 0.011 [-0.015, 0.043] c’: 0.236 [-0.006, 0.478]
	sdNFI-15	**a: 0.499 [0.121, 0.876]** **b: 0.064 [0.011, 0.116]** **ab: 0.032 [0.003, 0.076]** **c’: 0.333 [0.090, 0.576]**	**a: 0.560 [0.184, 0.935]** b: 0.010 [-0.062, 0.042] ab: 0.005 [-0.023, 0.037] c’: 0.238 [-0.003, 0.480]
Arithmetic fact retrieval		c**: 1.318 [0.505, 2.146]	c**: 0.752 [0.072, 1.449]
	sdNFI-7	**a: 0.578 [0.213, 0.943]** **b: 0.185 [0.033, 0.337]** **ab: 0.107 [0.010, 0.249]** **c’: 1.168 [0.449, 1.888]**	**a: 0.409 [0.038, 0.780]** b: -0.018 [-0.175, 0.140] ab: -0.007 [-0.075, 0.060] **c’: 0.820 [0.091, 1.549]**
	sdNFI-22	**a: 0.608 [0.215, 1.001]** **b: 0.184 [0.043, 0.325]** **ab: 0.112 [0.014, 0.258]** **c’: 1.169 [0.450, 1.888]**	**a: 0.518 [0.118, 0.917]** b: -0.004 [-0.150, 0.141] ab: -0.002 [-0.074, 0.074] **c’: 0.811 [0.082, 1.541]**
	sdNFI-28	**a: 0.510 [0.177, 0.843]** **b: 0.187 [0.023, 0.350]** **ab: 0.095 [0.003, 0.242]** **c’: 1.175 [0.456, 1.894]**	**a: 0.348 [0.010, 0.686]** b: 0.011 [-0.166, 0.188] ab: 0.004 [-0.059, 0.069] **c’: 0.813 [0.084, 1.542]**

*Beta coefficients (negative for F > M and positive for M > F) with 95% bootstrap CI; statistical significance is indicated in bold. **Descriptive total effect (not decomposed, see Methods) is indicated in italic

**Fig. 4:**
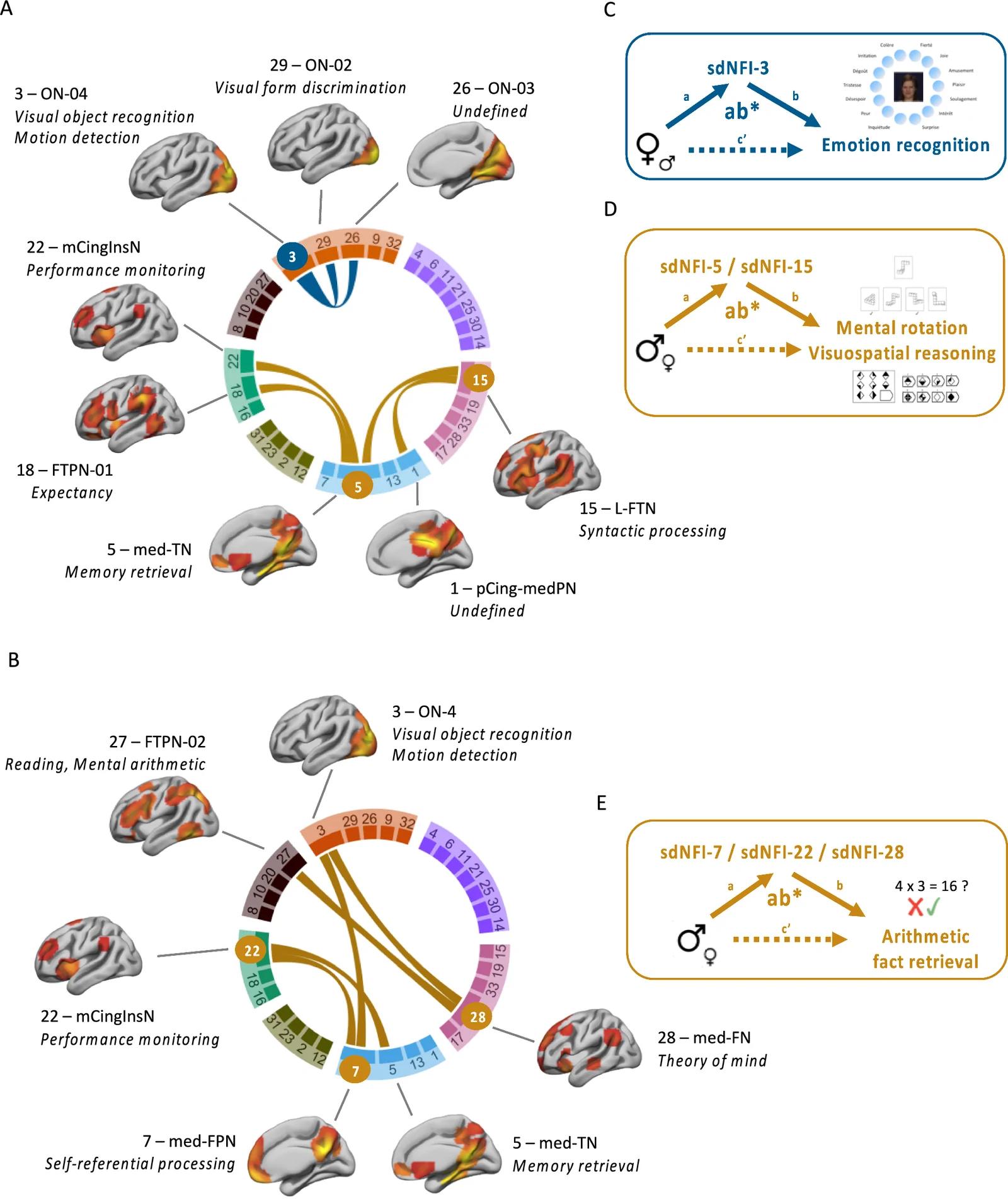
sdNFI-mediated S/G cognitive differences in declarative fields. **A**-**B**. Circos plots showing the sdNFIs involved. Numbers indicate GINNA networks (1–32); track colors denote functional modules (brown, cognitive flexibility; green, performance monitoring; orange, visual; pink, social/communication; turquoise, self-awareness). Link colors indicate direction of effects (beige, M > F; blue, F > M). sdNFIs are highlighted with circles. **C**-**E**. Moderated mediation models illustrating significant direct (c′) and component (a, b) paths (see Fig. 1). Significant indirect effects (a × b) are marked with * and were determined using bootstrap CIs. Abbreviations: a/m/p Cing, anterior/middle/posterior cingulate; F, frontal; Ins, insular; L/R, left/right; med, medial; N, network; O, occipital; P, parietal; T, temporal

In declarative fields (Fig. 4), some male sdNFIs mediated S/G differences in mental rotation, visuospatial reasoning, and arithmetic fact retrieval, while significant direct effects persisted, indicating partial mediation.

Male advantages in mental rotation and visuospatial reasoning were mediated by sdNFI-5 and sdNFI-15 (Fig. 4A). These sdNFIs linked regions across the performance monitoring, self-awareness, and social and communication modules, spanning function such as memory retrieval, expectancy, performance monitoring, and syntactic processing. Anatomically, sdNFI-5 integrated medial temporal (GINNA-5), fronto-temporo-parietal (GINNA-18), middle cingulo-insular (GINNA-22), and the left fronto-temporal (GINNA-15) networks. sdNFI-15 combined the left fronto-temporal (GINNA-15), medial temporal (GINNA-5), and posterior cingulate-medial parietal (GINNA-1) networks. All these networks are known to support the integration of spatial and memory-based representations [43].

Male-advantaged arithmetic fact retrieval performance was mediated by sdNFI-22, sdNFI-7, and sdNFI-28 (Fig. 4B). Together these sdNFIs formed a functional junction across multiple large-scale systems, including the performance monitoring, self-awareness, visual, social and communication, and cognitive flexibility modules, spanning processes such as memory retrieval, performance monitoring, self-referential integration, visual object processing, theory of mind, reading, and mental arithmetic. Anatomically, sdNFI-22 linked middle cingulo-insular (GINNA-22), medial temporal (GINNA-5), and medial fronto-parietal (GINNA-7) networks; sdNFI-7 connected medial fronto-parietal (GINNA-7) with medial temporal (GINNA-5), occipital (GINNA-3), and cingulo-insular (GINNA-22) networks; and sdNFI-28 integrated medial frontal (GINNA-28), occipital (GINNA-3), and fronto-temporo-parietal (GINNA-27) networks. The observed networks are in line with recent accounts of arithmetic fact retrieval as a distributed process engaging hippocampal memory structures, temporo-parietal language regions, and frontal control networks, along with perceptual processing areas [44, 45]. In contrast, calculation performance showed no significant S/G-mediated sdNFI effects in either knowledge domain [see Additional file 4].

**Fig. 5:**
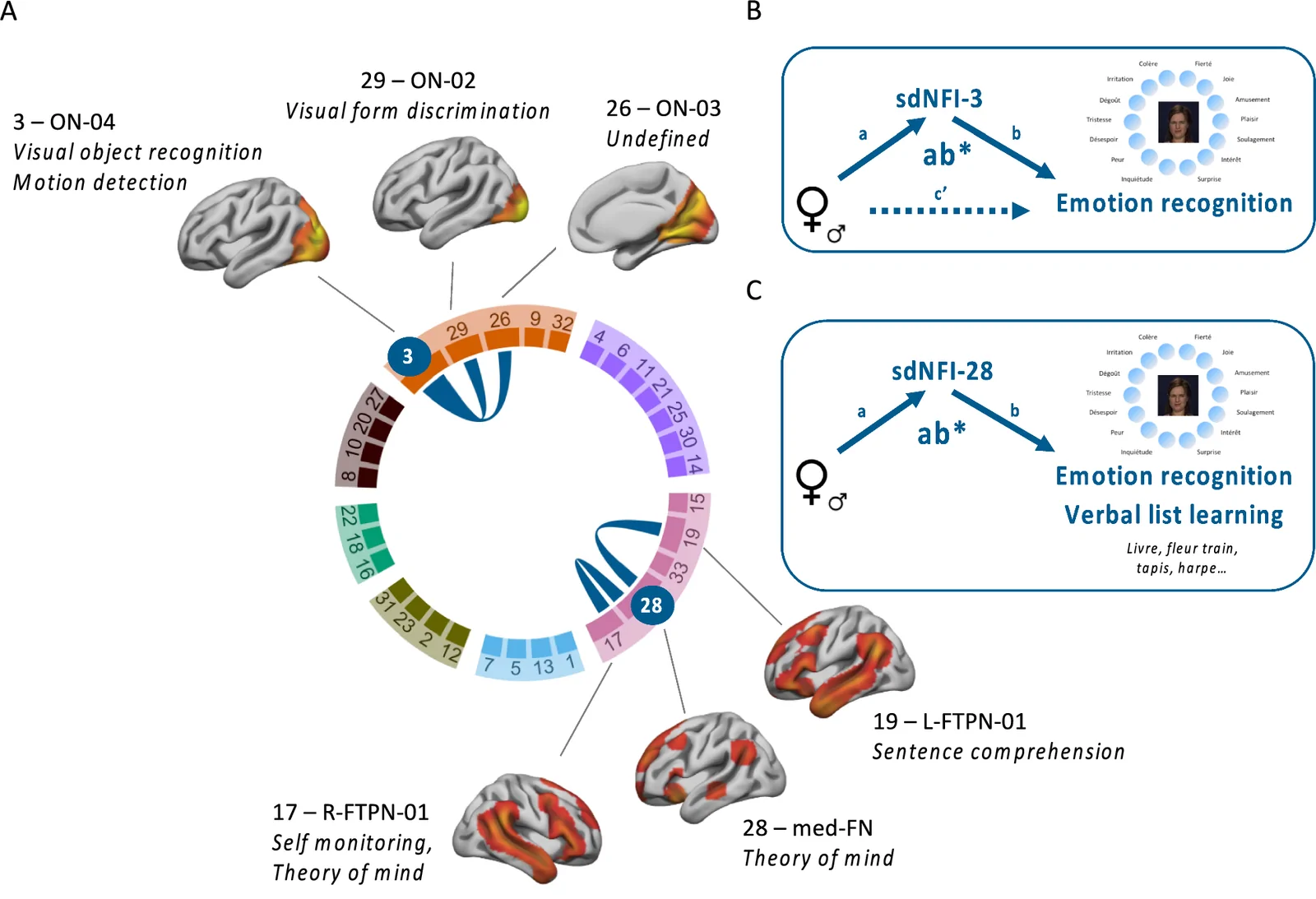
sdNFI-mediated S/G cognitive differences in situated fields. **A**. Circos plot showing the sdNFIs involved. Numbers indicate GINNA networks (1–32); track colors denote functional modules (pink, social/communication; orange, visual). Link color indicates direction of effects (blue, F > M). sdNFIs are highlighted with circles. **B**-**C**. Moderated mediation models illustrating significant direct (c′) and component (a, b) paths (see Fig. 1). Significant indirect effects (a × b) are marked with * and were determined using bootstrap CIs. Abbreviations: a/m/p Cing, anterior/middle/posterior cingulate; F, frontal; Ins, insular; L/R, left/right; med, medial; N, network; O, occipital; P, parietal; T, temporal

In women, emotion recognition and verbal list learning revealed a dissociable pattern of FC mediation across functional networks. For emotion recognition, sdNFI-3, composed of occipital visual networks (GINNA-3, GINNA-26, and GINNA-29), partially mediated female-advantaged performance across both declarative and situated fields (Figs. 4A, C, and 5). This pattern indicates a stable, context-independent contribution of visual networks to emotion recognition, consistent with prior neuroimaging evidence linking occipital and fusiform regions to the early visual analysis of facial features and basic emotion processing [46, 47]. No mediation effect involving sdNFI-3 was observed for verbal learning [see Additional file 4].

By contrast, sdNFI-28, spanning medial frontal and bilateral fronto-temporo-parietal networks involved in theory of mind, self-monitoring, and sentence comprehension (GINNA-28, GINNA-17, and GINNA-19), fully mediated S/G differences in emotion recognition and verbal list learning, but only in situated fields. This indicates complete, context-specific mediation, in line with evidence implicating the medial prefrontal cortex and temporo-parietal junction as core nodes of the mentalizing network supporting theory of mind, complex emotion recognition, and social-cognitive processing [48, 49].

Together, female patterns indicate that within and across cognitive tasks, distinct functional pathways support performance with different degrees of contextual sensitivity: lower-level visual networks provide stable support across environments, whereas higher-order socio-cognitive networks are selectively engaged as a function of academic training.

### FC-cognition associations are mainly bidirectional

Complementary models reversing the mediator and outcome were estimated to examine the directional specificity of the observed FC-cognition relationships. Most effects identified in the primary analyses persisted when cognitive performance was modeled as mediator and sdNFI as outcome [see Additional file 4], consistent with dynamic, bidirectional FC-cognition interactions. A notable exception was verbal list learning, for which the reversed model did not yield significant indirect effects in situated fields, suggesting that the FC-to-cognition direction is specific to this task and that the female advantage in verbal list learning is not reciprocally reflected in sdNFI-28 organization.

### sdNFI mediation varies as a function of academic stage within knowledge domains

**Fig. 6:**
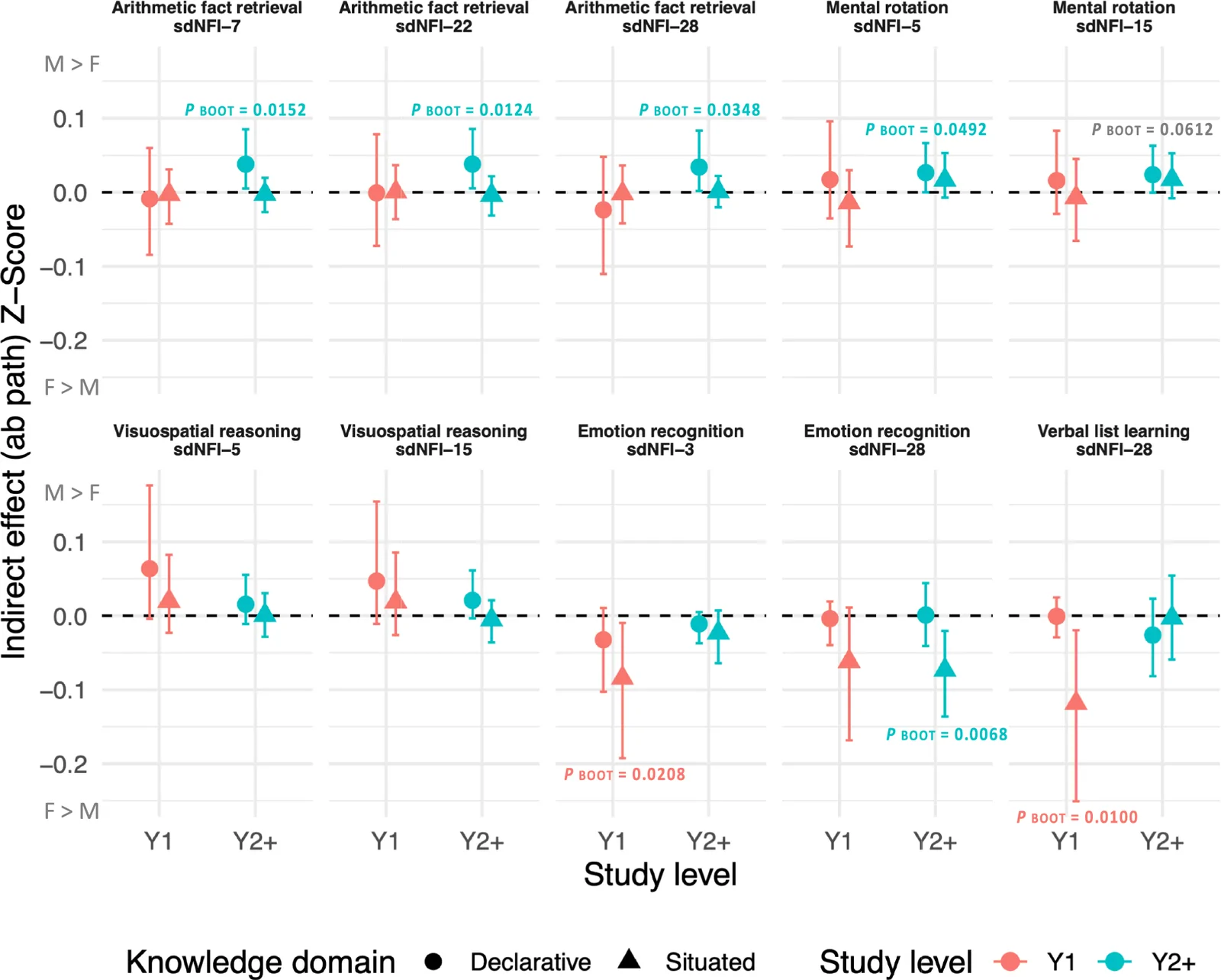
Academic stage as a moderator of sdNFI-mediated S/G differences in cognitive performance. Points represent the indirect effect (ab path) across study levels and knowledge domains, expressed as a Z-Score, with error bars indicating bootstrap 95% CIs. Colors distinguish study levels (Y1 in red and Y2 + in blue), and symbols represent knowledge domain (declarative and situated fields). Each panel corresponds to a specific combination of task and sdNFI dimension. Positive values indicate stronger effects for males than females (M > F), whereas negative values indicate stronger effects for females than males (F > M). Effects whose 95% CIs do not include zero are considered statistically significant. Bootstrap *P*-values (*P* BOOT) are displayed within panels to facilitate readability

To assess whether the observed mediation pathways were already present in first-year students or became more pronounced with subsequent academic experience, exploratory analyses incorporated study level (Y1 *vs* Y2 +) as an additional moderator of the conditional indirect effects (Fig. 6).

Results indicated a differential pattern of moderation by academic stage across cognitive tasks and sdNFIs. For arithmetic fact retrieval (sdNFI-7, sdNFI-22, and sdNFI-28 in declarative fields), mental rotation (sdNFI-5, with a trend for sdNFI-15 in declarative fields), and emotion recognition (sdNFI-28 in situated fields), moderated mediation effects were observed in Y2 + . In contrast, for emotion recognition (sdNFI-3) and verbal list learning (sdNFI-28) in situated fields, moderated mediation effects were present in Y1 but were not observed in Y2 + . No clear evidence of moderation by study level was found for visuospatial reasoning.

## Discussion

S/G differences in cognition are among the most robust findings in cognitive science, yet their neural implementation remains debated. In particular, it is unclear whether such differences primarily reflect stable properties of brain organization or are shaped by experience during periods of continued cognitive maturation. By integrating large-scale functional networks with university training in young adults, the present study shows that FC mediation of S/G cognitive differences is not uniform but depends jointly on task demands, functional network properties, and fields of academic training.

Across individuals, canonical FC-cognition relationships were preserved and mapped onto broad cognitive domains rather than task-specific processes, consistent with evidence that intrinsic FC captures domain-general cognitive organization [50, 51]. In the current study, domain-level associations manifested as visuospatial and arithmetic fact retrieval abilities in men, supported by parietal and medial temporal regions, and communicative abilities in women, supported by medial prefrontal and temporo-parietal networks. Crucially, the expression of these associations, both in magnitude and in mediation structure, was strongly contingent on academic training fields (Figs. 4 and 5), despite comparable S/G differences in both cognitive performance and FC across knowledge domains. This dissociation suggests that higher education does not significantly alter baseline abilities, which can emerge early in schooling as shown for instance for mathematical performance [52]. Here, academic-domain differences were expressed less in cognitive performance itself than in the neural architecture associated with that performance. Importantly, this architecture varied across academic stages (Fig. 6), with several FC-mediated effects observed primarily at later stages of university training and others attenuating over time. Together, these findings suggest that educational experience shapes the neural substrates through which cognitive performance is achieved. Educational experience is known to influence the selection and deployment of learning and metacognitive strategies [53]. The present findings suggest that such experience-dependent processes may be reflected in the coupling between functional networks and task performance.

In this view, FC-cognition relationships may index the engagement of experience-relevant networks rather than baseline ability per se, in line with evidence that individual differences in FC predict distinct decision strategies independently of overall task accuracy [54]. The bidirectional nature of the FC-cognition relationships, as indicated by the reverse mediation analyses, further supports a co-evolutionary dynamic in which network organization both reflects and adapts to the cognitive demands of academic training, echoing the concept of adaptive dynamical networks in complex systems [55]. Verbal list learning presented a theoretically informative exception: the absence of significant reverse mediation effects, together with the presence of FC-mediated effects at Y1 but not at Y2 + , suggests attenuation of pre-existing FC-cognition coupling across academic stages.

Taken together, the observed variations associated with university training across knowledge domains and academic stages may reflect heterogeneous changes in FC-cognition associations, consistent with experience-dependent brain plasticity and network reconfiguration mechanisms [19].

Beyond S/G-specific patterns of moderated mediation, we identified situations in which academic training fields exerted little or no influence on FC-cognition relationships. Two distinct mechanisms appeared to underlie this lack of contextual modulation. First, when low-level perceptual networks were engaged in task performance, educational effects were negligible. Emotion recognition illustrates this pattern: visual networks connectivity mediated S/G differences in performance consistently across knowledge domains, indicating largely context-independent FC-cognition coupling. This aligns with evidence that emotionally expressive faces reliably engage early visual cortex [56] and that stable visual-to-higher-order networks support emotion decoding [48]. Second, for tasks engaging higher-order, distributed networks that afford multiple solution strategies, educational modulation was also absent. Calculation exemplifies this case: no FC mediation was detected in either knowledge domain, likely reflecting heterogeneity in the functional networks recruited across individuals due to variable strategy use [57, 58]. Together, these findings suggest that higher education influences cognition indirectly by biasing strategy selection and stabilizing the engagement of specific functional networks, with effects that depend on both S/G and task characteristics.

Several limitations should be acknowledged. First, because candidate networks were selected on the basis of sex-related variation observed in the present sample, subsequent mediation analyses should be interpreted as exploratory. Although the identified connectivity patterns converged with prior literature [40, 41, 42], independent replication using a priori network selection and/or independent validation samples will be necessary to establish the robustness and generalizability of the proposed mechanisms. Second, the cross-sectional design precludes direct causal inference. The study-level analyses provide preliminary evidence regarding the timing of FC-cognition mediation effects across academic stage, suggesting that some pathways may already be present at university entry whereas others appear at later stages of training. These hypotheses will require confirmation in longitudinal studies following the same individuals across the course of their academic development. Third, generalizability may be limited. The i-Share cohort consists of young French university students, and the observed patterns may not extend to other cultural, educational, or age contexts in which the declarative/situated distinction maps differently onto disciplinary structures. Finally, knowledge domain was operationalized based on students’ declared field of study, without direct measurement of exposure to specific epistemic practices or learning strategies. Individual variability within fields, in terms of teaching approaches, social engagement, or practical training, may introduce classification noise that attenuates observed effects.

### Perspectives and significance

The present findings open several avenues for future research. Longitudinal designs would allow direct testing of whether the temporal sequences observed here reflect a causal consolidation/dissolution process driven by academic exposure. Beyond higher education, the framework examining S/G cognitive differences through the lens of experience-dependent network recruitment, rather than binary group comparisons, could be extended to other structured environmental contexts in which sociocultural exposure systematically differs. More broadly, the demonstration that the same S/G cognitive differences are expressed through distinct network pathways depending on the epistemic nature of training suggests that brain-behavior relationships are not fixed properties of biological sex but contingent outcomes of the interaction between intrinsic network architecture and accumulated experience. This has implications for educational practice: if network recruitment patterns are shaped by the type of knowledge emphasized during training, then pedagogical approaches that deliberately integrate declarative and situated learning dimensions may foster more flexible cognitive profiles across S/G groups.

## Conclusions

By leveraging academic training as a naturalistic source of environmental variation, this study demonstrates that S/G differences in cognitive performance are preserved across knowledge domains, while the FC pathways mediating these differences are selectively organized by the epistemic nature of university training and evolve across academic stage. Declarative and situated knowledge domains recruit distinct, context-sensitive networks to support S/G-advantaged performance, without altering baseline cognitive profiles. These findings provide a mechanistic account of how higher education shapes the brain architecture supporting S/G cognitive differences during early adulthood, and highlight the importance of moving beyond static, binary frameworks toward a dynamic, context-sensitive understanding of gendered brain organization.

## Supplementary Information

Additional file 1.

Additional file 2.

Additional file 3.

Additional file 4.

## Data Availability

The datasets generated and analyzed during the current study are not publicly available as they contain information that could compromise research participant consent. However, data are available from the corresponding author upon reasonable request and with permission of the principal investigator of the i-Share project.
