# Supplementary material for: Academic training selectively shapes the neural pathways mediating sex/gender differences in cognition
Source: Biol Sex Differ. 2026 Aug 6;17:175. doi: 10.1186/s13293-026-00963-0 (PMC13733161; doi:10.1186/s13293-026-00963-0)
Supplement: Supplementary file 1 [file 13293_2026_963_MOESM1_ESM.pdf]

Additional Table 1. Distribution of S/G across academic domains and cognitive task subsamples.

Sample	Field	N_total	N_female	Pct_female	N_male	Pct_male
Full sample	Declarative	843	607	72.0	236	28.0
Full sample	Situated	858	615	71.7	243	28.3
Arithmetic fact retrieval	Declarative	636	477	75.0	159	25.0
Arithmetic fact retrieval	Situated	609	450	73.9	159	26.1
Calculation	Declarative	598	442	73.9	156	26.1
Calculation	Situated	595	442	74.3	153	25.7
Emotion recognition	Declarative	696	503	72.3	193	27.7
Emotion recognition	Situated	565	414	73.3	151	26.7
Mental rotation	Declarative	641	483	75.4	158	24.6
Mental rotation	Situated	603	443	73.5	160	26.5
Visuospatial reasoning	Declarative	580	435	75.0	145	25.0
Visuospatial reasoning	Situated	578	425	73.5	153	26.5
Verbal list learning	Declarative	657	478	72.8	179	27.2
Verbal list learning	Situated	543	398	73.3	145	26.7
