# Supplementary material for: Academic training selectively shapes the neural pathways mediating sex/gender differences in cognition
Source: Biol Sex Differ. 2026 Aug 6;17:175. doi: 10.1186/s13293-026-00963-0 (PMC13733161; doi:10.1186/s13293-026-00963-0)
Supplement: Supplementary file 2 [file 13293_2026_963_MOESM2_ESM.pdf]

Additional Fig. 1. Selection of S/G-associated functional connectivity (FC) edges.

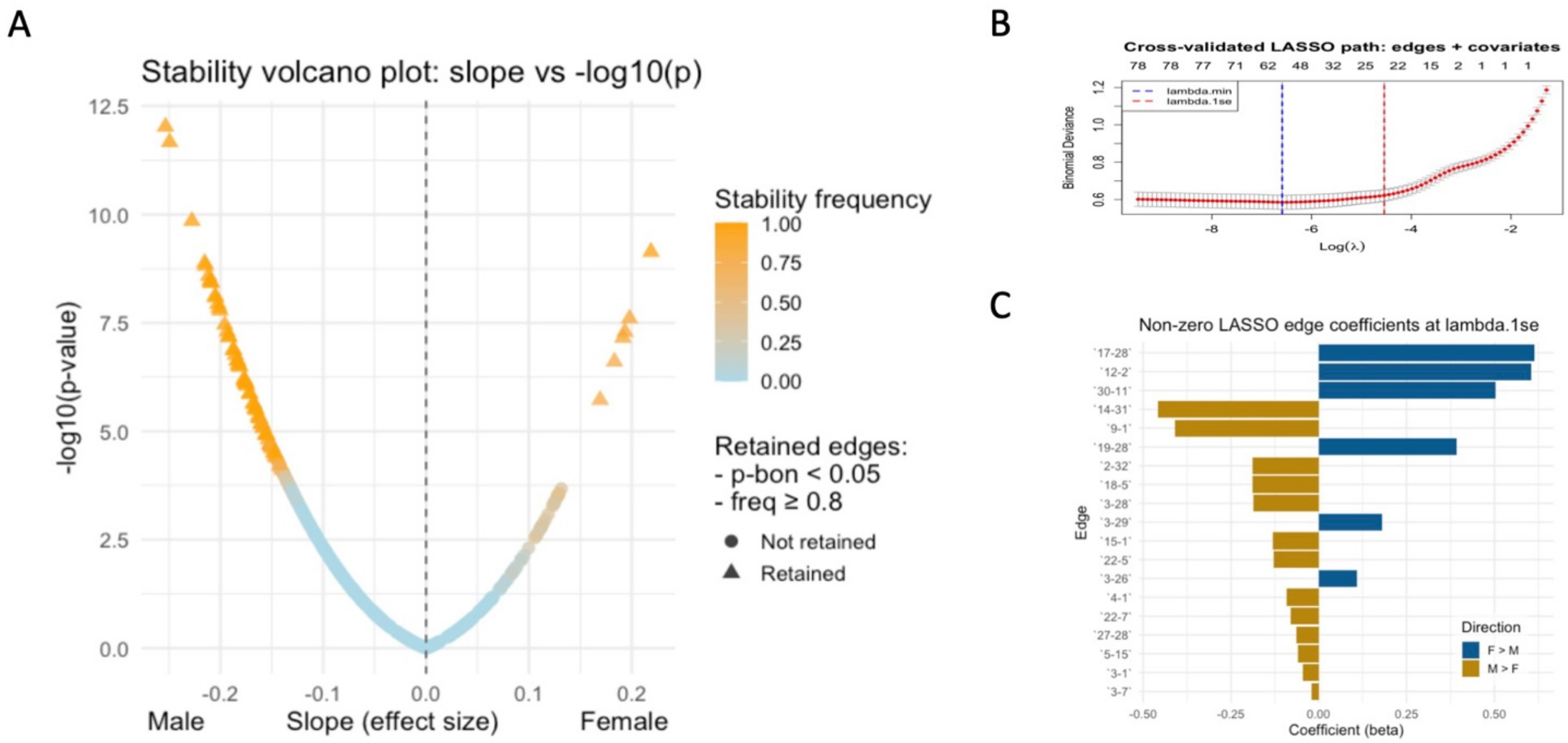

A. Edges were pre-selected using linear mixed-effects modeling and nested cross-validation, followed by stability selection (frequency ≥ 0.8, Bonferroni-corrected $P < 0.05$), yielding 72 edges. **B-C.** LASSO logistic regression (λ = 1) retained 19 edges with non-zero coefficients predicting S/G after covariate adjustment.
