# Supplementary material for: Academic training selectively shapes the neural pathways mediating sex/gender differences in cognition
Source: Biol Sex Differ. 2026 Aug 6;17:175. doi: 10.1186/s13293-026-00963-0 (PMC13733161; doi:10.1186/s13293-026-00963-0)
Supplement: Supplementary file 3 [file 13293_2026_963_MOESM3_ESM.pdf]

Additional Table 2. S/G specific sdNFIs (sex-differential nodal FC indices) based on GINNA.

Female

**sdNFI**	**GINNA network**	**Anatomic labeling**	**Cognitive labeling**
**3**	3	Occipital Network-04	Visual object recognition, motion detection
	26	Occipital Network-03	-
	29	Occipital Network-02	Visual form discrimination
**28**	28	Medial frontal network	Theory of mind
	17	Right fronto-temporo-parietal network-01	Self-monitoring, theory of mind
	19	Left fronto-temporo-parietal network-01	Sentence comprehension

Male

**sdNFI**	**GINNA network**	**Anatomic labeling**	**Cognitive labeling**
**1**	1	Posterior cingulate - medial parietal network	-
	3	Occipital Network-04	Visual object recognition, motion detection
	4	Temporal network-01	Articulation
	9	Occipital Network-01	Visual perception
**3**	3	Occipital Network -04	Visual object recognition, motion detection
	1	Posterior cingulate - medial parietal network	-
	7	Medial fronto-parietal network	Self-referential processing
	28	Medial frontal network	Theory of mind
**5**	5	Medial temporal network	Memory retrieval
	15	Left fronto-temporal network	Syntactic processing
	18	Fronto-temporo-parietal network-01	Expectancy
	22	Medial cingulate - insular network	Performance monitoring
**7**	7	Medial fronto-parietal network	Self-referential processing
	3	Occipital Network-04	Visual object recognition, motion detection
	22	Medial cingulate - insular network	Performance monitoring
**15**	15	Left fronto-temporal network	Syntactic processing
	1	Posterior cingulate - medial parietal network	-
**22**	22	Medial cingulate - insular network	Performance monitoring
	5	Medial temporal network	Memory retrieval
	7	Medial fronto-parietal network	Self-referential processing
	15	Left fronto-temporal network	Syntactic processing
**28**	28	Medial frontal network	Theory of mind
	3	Occipital Network-04	Visual object recognition, motion detection
	27	Left fronto-temporo-parietal network-02	Reading, Mental arithmetic
