# Supplementary material for: Academic training selectively shapes the neural pathways mediating sex/gender differences in cognition
Source: Biol Sex Differ. 2026 Aug 6;17:175. doi: 10.1186/s13293-026-00963-0 (PMC13733161; doi:10.1186/s13293-026-00963-0)
Supplement: Supplementary file 4 [file 13293_2026_963_MOESM4_ESM.pdf]

**Additional Table 3. Moderated direct (c′) and indirect (ab) S/G effects in sdNFI → Cognition and Cognition → sdNFI mediation models*.**

| **Knowledge domain:** | | **Declarative fields** | | **Situated fields** | |
|---|---|---|---|---|---|
| **Mediation pathway:** | | **S/G → sdNFI → Cognition** | **S/G → Cognition → sdNFI** | **S/G → sdNFI → Cognition** | **S/G → Cognition → sdNFI** |
| ***Female advantaged-performance*** | | | | | |
| Emotion recognition | sdNFI-3 | **ab: -0.0686 [-0.1805, -0.0003]**<br>**c': -1.1363 [-1.9009, -0.3718]** | **ab: -0.0402 [-0.0975, -0.0030]**<br>**c': -0.3199 [-0.6372, -0.0026]** | **ab: -0.1561 [-0.3202, -0.0303]**<br>**c': -0.9841 [-1.8335, -0.1348]** | **ab: -0.0559 [-0.1273, -0.0074]**<br>**c': -0.4790 [-0.8295, -0.1285]** |
| | sdNFI-28 | ab: -0.0035 [-0.1218, 0.1127]<br>**c': -1.2298 [-2.0010, -0.4586]** | ab: -0.0021 [-0.0342, 0.0301]<br>**c': -0.4125 [-0.6483, -0.1768]** | **ab: -0.2862 [-0.5193, -0.1051]**<br>c': -0.8582 [-1.7177, 0.0013] | **ab: -0.0468 [-0.1014, -0.0091]**<br>**c': -0.5996 [-0.8600, -0.3392]** |
| Verbal list learning | sdNFI-3 | ab: -0.0472 [-0.1346, 0.0093]<br>**c': -0.9153 [-1.5814, -0.2491]** | ab: -0.0290 [-0.0822, 0.0059]<br>**c': -0.3357 [-0.6640, -0.0075]** | ab: -0.0360 [-0.1408, 0.0600]<br>c': -0.2349 [-0.9687, 0.4990] | ab: -0.0049 [-0.0346, 0.0136]<br>**c': -0.5317 [-0.8903, -0.1732]** |
| | sdNFI-28 | ab: -0.0515 [-0.1706, 0.0523]<br>**c': -0.9260 [-1.5989, -0.2532]** | ab: -0.0131 [-0.0492, 0.0141]<br>**c': -0.4705 [-0.7142, -0.2268]** | **ab: -0.1692 [-0.3581, -0.0207]**<br>c': -0.1003 [-0.8412, 0.6405] | ab: -0.0097 [-0.0447, 0.0175]<br>**c': -0.6294 [-0.8956, -0.3631]** |
| ***Male advantaged-performance*** | | | | | |
| Mental rotation | sdNFI-1 | ab: 0.0102 [-0.0238, 0.0520]<br>**c': 0.3534 [0.0872, 0.6196]** | ab: 0.0139 [-0.0371, 0.0722]<br>**c': 0.7371 [0.2697, 1.2045]** | ab: 0.0195 [-0.0195, 0.0623]<br>c': 0.1886 [-0.0826, 0.4599] | ab: 0.0158 [-0.0172, 0.0645]<br>**c': 0.8173 [0.3440, 1.2907]** |
| | sdNFI-3 | ab: 0.0138 [-0.0169, 0.0520]<br>**c': 0.3510 [0.0849, 0.6170]** | ab: 0.0236 [-0.0277, 0.0881]<br>**c': 0.6650 [0.1910, 1.1391]** | ab: 0.0140 [-0.0145, 0.0473]<br>c': 0.1930 [-0.0778, 0.4638] | ab: 0.0143 [-0.0163, 0.0616]<br>**c': 0.6109 [0.1308, 1.0910]** |
| | sdNFI-5 | **ab: 0.0327 [0.0027, 0.0791]**<br>**c': 0.3368 [0.0714, 0.6022]** | **ab: 0.0688 [0.0057, 0.1648]**<br>**c': 0.7790 [0.1828, 1.3751]** | ab: 0.0120 [-0.0188, 0.0477]<br>c': 0.1935 [-0.0768, 0.4638] | ab: 0.0142 [-0.0256, 0.0710]<br>**c': 0.8533 [0.2497, 1.4570]** |
| | sdNFI-7 | ab: 0.0278 [-0.0021, 0.0715]<br>**c': 0.3391 [0.0735, 0.6048]** | ab: 0.0355 [-0.0026, 0.0922]<br>**c': 0.4952 [0.1260, 0.8644]** | ab: 0.0109 [-0.0106, 0.0400]<br>c': 0.1954 [-0.0743, 0.4650] | ab: 0.0117 [-0.0119, 0.0496]<br>**c': 0.3741 [0.0002, 0.7480]** |
| | sdNFI-15 | **ab: 0.0294 [0.0009, 0.0755]**<br>**c': 0.3394 [0.0742, 0.6045]** | **ab: 0.0401 [0.0018, 0.0959]**<br>**c': 0.4320 [0.0673, 0.7967]** | ab: 0.0168 [-0.0157, 0.0561]<br>c': 0.1876 [-0.0826, 0.4579] | ab: 0.0121 [-0.0138, 0.0503]<br>**c': 0.5601 [0.1908, 0.9204]** |
| | sdNFI-22 | ab: 0.0286 [-0.0004, 0.0727]<br>**c': 0.3410 [0.0755, 0.6060]** | ab: 0.0396 [-0.0007, 0.1011]<br>**c': 0.5241 [0.1260, 0.9221]** | ab: 0.0084 [-0.0208, 0.0408]<br>c': 0.1967 [-0.0734, 0.4698] | ab: 0.0076 [-0.0198, 0.0446]<br>**c': 0.5272 [0.1241, 0.9303]** |
| | sdNFI-28 | ab: 0.0120 [-0.0153, 0.0476]<br>**c': 0.3531 [0.0873, 0.6190]** | ab: 0.0155 [-0.0191, 0.0600]<br>**c': 0.4579 [0.1220, 0.7937]** | ab: 0.0073 [-0.0158, 0.0336]<br>c': 0.1998 [-0.0778, 0.4698] | ab: 0.0067 [-0.0156, 0.0381]<br>c': 0.3217 [-0.0184, 0.6618] |
| Visuospatial reasoning | sdNFI-1 | ab: 0.0081 [-0.0239, 0.0462]<br>**c': 0.3520 [0.1081, 0.5959]** | ab: 0.0159 [-0.0459, 0.0846]<br>**c': 0.7257 [0.2355, 1.2159]** | ab: 0.0001 [-0.0309, 0.0309]<br>**c': 0.2473 [0.0049, 0.4899]** | ab: -0.0009 [-0.0489, 0.0433]<br>**c': 0.7617 [0.2782, 1.2451]** |
| | sdNFI-3 | ab: 0.0132 [-0.0158, 0.0517]<br>**c': 0.3466 [0.1026, 0.5905]** | ab: 0.0266 [-0.0315, 0.0993]<br>**c': 0.7202 [0.2224, 1.2180]** | ab: 0.0023 [-0.0256, 0.0294]<br>**c': 0.2462 [0.0039, 0.4886]** | ab: 0.0030 [-0.0447, 0.0512]<br>**c': 0.6298 [0.1389, 1.1208]** |
| | sdNFI-5 | **ab: 0.0319 [0.0025, 0.0744]**<br>**c': 0.3305 [0.0874, 0.5736]** | **ab: 0.0851 [0.0071, 0.1988]**<br>**c': 0.7954 [0.1716, 1.4193]** | ab: 0.0108 [-0.0149, 0.0427]<br>c': 0.2360 [-0.0057, 0.4777] | ab: 0.0185 [-0.0353, 0.0880]<br>**c': 0.8249 [0.2096, 1.4402]** |
| | sdNFI-7 | ab: 0.0239 [-0.0039, 0.0665]<br>**c': 0.3374 [0.0939, 0.5809]** | ab: 0.0383 [-0.0053, 0.1020]<br>**c': 0.5127 [0.1256, 0.8997]** | ab: 0.0039 [-0.0184, 0.0283]<br>**c': 0.2430 [0.0016, 0.4844]** | ab: 0.0055 [-0.0291, 0.0460]<br>**c': 0.3971 [0.0153, 0.7788]** |
| | sdNFI-15 | **ab: 0.0319 [0.0027, 0.0759]**<br>**c': 0.3329 [0.0901, 0.5756]** | **ab: 0.0554 [0.0067, 0.1255]**<br>**c': 0.4272 [0.0451, 0.8094]** | ab: 0.0054 [-0.0226, 0.0375]<br>c': 0.2382 [-0.0034, 0.4799] | ab: 0.0047 [-0.0307, 0.0428]<br>**c': 0.5619 [0.1850, 0.9388]** |
| | sdNFI-22 | ab: 0.0256 [-0.0019, 0.0668]<br>**c': 0.3376 [0.0944, 0.5808]** | ab: 0.0468 [-0.0022, 0.1170]<br>**c': 0.5138 [0.0988, 0.9288]** | ab: 0.0027 [-0.0225, 0.0303]<br>**c': 0.2433 [0.0018, 0.4849]** | ab: 0.0076 [-0.0198, 0.0446]<br>**c': 0.4970 [0.0877, 0.9063]** |
| | sdNFI-28 | ab: 0.0186 [-0.0075, 0.0579]<br>**c': 0.3435 [0.1001, 0.5868]** | ab: 0.0302 [-0.0103, 0.0868]<br>**c': 0.4443 [0.0921, 0.7965]** | ab: 0.0022 [-0.0281, 0.0185]<br>**c': 0.2498 [0.0083, 0.4913]** | ab: 0.0043 [-0.0409, 0.0264]<br>c': 0.3447 [-0.0026, 0.6921] |
| Arithmetic fact retrieval | sdNFI-1 | ab: 0.0303 [-0.0712, 0.1498]<br>**c': 1.2331 [0.5114, 1.9548]** | ab: 0.0106 [-0.0451, 0.0752]<br>**c': 0.7996 [0.3320, 1.2671]** | ab: -0.0148 [-0.1098, 0.0776]<br>**c': 0.8281 [0.0946, 1.5617]** | ab: -0.0019 [-0.0517, 0.0501]<br>**c': 0.8246 [0.3516, 1.2976]** |
| | sdNFI-3 | ab: 0.0676 [-0.0290, 0.1960]<br>**c': 1.1964 [0.4755, 1.9173]** | ab: 0.0372 [-0.0210, 0.1148]<br>**c': 0.7207 [0.2454, 1.1960]** | ab: -0.0043 [-0.0837, 0.0765]<br>**c': 0.8256 [0.0931, 1.5582]** | ab: 0.0017 [-0.0483, 0.0544]<br>**c': 0.6676 [0.1867, 1.1484]** |
| | sdNFI-5 | ab: 0.0831 [-0.0085, 0.2158]<br>**c': 1.1923 [0.4727, 1.9118]** | ab: 0.0600 [-0.0124, 0.1639]<br>**c': 0.8504 [0.2536, 1.4472]** | ab: -0.0121 [-0.0900, 0.0646]<br>**c': 0.8257 [0.0931, 1.5568]** | ab: -0.0030 [-0.0655, 0.0641]<br>**c': 0.8302 [0.2265, 1.4339]** |
| | sdNFI-7 | **ab: 0.1068 [0.0095, 0.2490]**<br>**c': 1.1682 [0.4489, 1.8875]** | **ab: 0.0484 [0.0030, 0.1177]**<br>**c': 0.5236 [0.1550, 0.8922]** | ab: -0.0072 [-0.0754, 0.0605]<br>**c': 0.8195 [0.0906, 1.5486]** | ab: -0.0008 [-0.0391, 0.0413]<br>**c': 0.4137 [0.0408, 0.7865]** |
| | sdNFI-15 | ab: 0.0537 [-0.0312, 0.1744]<br>**c': 1.2227 [0.5034, 1.9421]** | ab: 0.0251 [-0.0185, 0.0828]<br>**c': 0.4890 [0.1251, 0.8530]** | ab: -0.0343 [-0.1280, 0.0465]<br>**c': 0.8406 [0.1091, 1.5720]** | ab: -0.0133 [-0.0572, 0.0256]<br>**c': 0.5645 [0.1962, 0.9327]** |
| | sdNFI-22 | **ab: 0.1119 [0.0141, 0.2580]**<br>**c': 1.1690 [0.4504, 1.8876]** | **ab: 0.0544 [0.0056, 0.1277]**<br>**c': 0.5480 [0.1513, 0.9448]** | ab: -0.0023 [-0.0736, 0.0740]<br>**c': 0.8113 [0.0818, 1.5409]** | ab: 0.0041 [-0.0352, 0.0507]<br>**c': 0.5174 [0.1161, 0.9188]** |
| | sdNFI-28 | **ab: 0.0951 [0.0026, 0.2425]**<br>**c': 1.1753 [0.4561, 1.8945]** | **ab: 0.0442 [0.0016, 0.1070]**<br>**c': 0.4610 [0.1253, 0.7968]** | ab: 0.0039 [-0.0591, 0.0692]<br>**c': 0.8132 [0.0840, 1.5425]** | ab: 0.0035 [-0.0300, 0.0413]<br>**c': 0.3472 [0.0078, 0.6869]** |
| Calculation | sdNFI-1 | ab: -0.0130 [-0.0612, 0.0350]<br>**c': 0.6659 [0.3717, 0.9601]** | ab: -0.0203 [-0.1072, 0.0640]<br>**c': 0.8731 [0.3942, 1.3520]** | ab: -0.0009 [-0.0413, 0.0413]<br>**c': 0.5036 [0.2062, 0.8011]** | ab: -0.0050 [-0.0803, 0.0699]<br>**c': 0.8022 [0.3182, 1.2862]** |
| | sdNFI-3 | ab: -0.0049 [-0.0451, 0.0376]<br>**c': 0.6605 [0.3666, 0.9545]** | ab: -0.0095 [-0.0983, 0.0767]<br>**c': 0.7885 [0.3004, 1.2766]** | ab: -0.0084 [-0.0450, 0.0261]<br>**c': 0.5098 [0.2124, 0.8071]** | ab: -0.0196 [-0.1014, 0.0546]<br>**c': 0.6715 [0.1782, 1.1648]** |
| | sdNFI-5 | ab: 0.0068 [-0.0327, 0.0502]<br>**c': 0.6527 [0.3592, 0.9462]** | ab: 0.0212 [-0.0843, 0.1339]<br>**c': 0.9075 [0.2964, 1.5186]** | ab: -0.0130 [-0.0509, 0.0178]<br>**c': 0.5135 [0.2166, 0.8104]** | ab: -0.0396 [-0.1438, 0.0454]<br>**c': 0.8393 [0.2216, 1.4569]** |
| | sdNFI-7 | ab: 0.0118 [-0.0243, 0.0553]<br>**c': 0.6475 [0.3540, 0.9409]** | ab: 0.0224 [-0.0432, 0.0946]<br>**c': 0.5378 [0.1590, 0.9165]** | ab: -0.0094 [-0.0429, 0.0176]<br>**c': 0.5096 [0.2134, 0.8059]** | ab: -0.0220 [-0.0866, 0.0343]<br>**c': 0.4197 [0.0368, 0.8025]** |
| | sdNFI-15 | ab: 0.0025 [-0.0353, 0.0451]<br>**c': 0.6547 [0.3614, 0.9480]** | ab: 0.0084 [-0.0575, 0.0774]<br>**c': 0.5182 [0.1460, 0.8905]** | ab: -0.0070 [-0.0450, 0.0303]<br>**c': 0.5069 [0.2098, 0.8040]** | ab: -0.0145 [-0.0741, 0.0411]<br>**c': 0.5700 [0.1937, 0.9462]** |
| | sdNFI-22 | ab: 0.0102 [-0.0255, 0.0525]<br>**c': 0.6510 [0.3578, 0.9442]** | ab: 0.0203 [-0.0497, 0.0978]<br>**c': 0.5709 [0.1635, 0.9783]** | ab: -0.0142 [-0.0510, 0.0135]<br>**c': 0.5138 [0.2116, 0.8103]** | ab: 0.0303 [-0.1001, 0.0293]<br>**c': 0.5197 [0.1079, 0.9314]** |
| | sdNFI-28 | ab: -0.0062 [-0.0427, 0.0326]<br>**c': 0.6619 [0.3685, 0.9553]** | ab: -0.0084 [-0.0721, 0.0546]<br>**c': 0.5116 [0.1669, 0.8563]** | ab: -0.0068 [-0.0386, 0.0197]<br>**c': 0.5080 [0.2116, 0.8044]** | ab: -0.0160 [-0.0727, 0.0350]<br>**c': 0.3570 [0.0085, 0.7054]** |

*Beta coefficients (positive for M > F and negative for F > M) with 95% bootstrap CI; significance is indicated in bold.
